# Novel lysine‐specific histone demethylase 1 inhibitor in acute myeloid leukaemia transformed from essential thrombocythaemia

**DOI:** 10.1002/cnr2.1588

**Published:** 2021-11-16

**Authors:** Samantha Hodges, Julian Cooney

**Affiliations:** ^1^ Fiona Stanley Hospital Murdoch Western Australia Australia; ^2^ PathWest Laboratory Medicine Nedlands Western Australia Australia; ^3^ University of Western Australia Perth Western Australia Australia

**Keywords:** AML transformed from ET, Bomedemstat, LSD1 inhibitor, Novel AML therapies

## Abstract

**Background:**

While progress continues in the understanding of molecular abnormalities in acute myeloid leukaemia (AML), with some specific targeted therapies now available, it remains commonly fatal in the elderly. Leukaemic evolution and transformation from myeloproliferative neoplasms (MPN) may be associated with increased numbers of mutations in the genes associated with myeloid neoplasm and the prognosis in such patients is invariably dismal. Targeting of intracellular enzymes associated with integral cellular function has advanced understanding and promises improvements in treatments.

**Case:**

We report impressive prolonged response to therapy in a case of secondary AML, arising from essential thrombocythaemia (ET). The trial agent, the oral lysine‐specific histone demethylase 1 (LSD1) inhibitor Bomedemstat (IMG‐7289) was well tolerated. In addition to suppressing the malignant clone, these blasts showed differentiation to monocytes morphologically as well as by surface markers seen on flow cytometry. Bomedemstat has efficacy in the treatment of myelofibrosis and may have a special role in treatment of specific AML subtypes, including secondary leukaemias arising from MPN as seen.

**Conclusion:**

We report a case of an older adult with secondary AML transformed from ET, with a remarkable response to LSD1 inhibition with Bomedemstat, with prolonged reduction in blasts demonstrating differentiation to monocytes.

## 1 INTRODUCTION

While progress continues in the understanding of molecular abnormalities in acute myeloid leukaemia (AML), with some specific targeted therapies now available, it remains commonly fatal in the elderly.^1^ Leukaemic evolution and transformation from myeloproliferative neoplasms (MPN) may be associated with increased numbers of mutations in the genes associated with myeloid neoplasm and the prognosis in such patients is invariably dismal.^1^, ^2^ Targeting of intracellular enzymes associated with integral cellular function has advanced understanding and promises improvements in treatments. We report impressive prolonged response to therapy in a case of secondary AML, arising from essential thrombocythaemia (ET). The trial agent, the oral lysine‐specific histone demethylase 1 (LSD1) inhibitor Bomedemstat (IMG‐7289) was well tolerated. In addition to suppressing the malignant clone, these blasts showed differentiation to monocytes morphologically as well as by surface markers seen on flow cytometry. Bomedemstat has efficacy in the treatment of myelofibrosis and may have a special role in treatment of specific AML subtypes, including secondary leukaemias arising from MPN as seen. We report a case of an older adult with secondary AML transformed from ET, with a remarkable response to LSD1 inhibition with Bomedemstat, with prolonged reduction in blasts demonstrating differentiation to monocytes.

## 2 CASE DESCRIPTION

An 84‐year‐old female with a 16‐year history of Janus Kinase‐2 (JAK‐2) positive ET presented with a lower respiratory tract infection and was found to have pancytopenia with occasional circulating blasts. Previous therapies included busulfan and hydroxycarbamide. She then became transfusion dependent and blast counts increased rapidly. Bone marrow biopsy showed a hypercellular marrow with markedly reduced trilineage haematopoiesis and 65% myeloblasts, with mildly increased reticulin deposition. Cytogenetics showed a normal karyotype 46XX. Molecular studies found no *JAK2*, Calreticulin (*CALR*) or Myeloproliferative Leukaemia Gene (*MPL*) mutations or variants, however did show two somatic mutations in DNA (cytosine‐5)‐methyltransferase 3A (*DNMT3A*) in *trans*: G707S missense mutation and H506P missense mutation.

**FIGURE 1:**
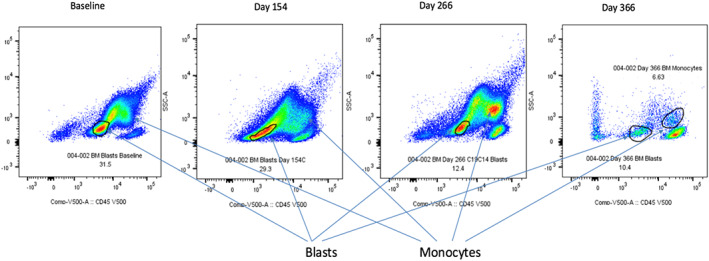
Progressive differentiation of blasts to monocytes (SCC/CD45 gating)

**FIGURE 2:**
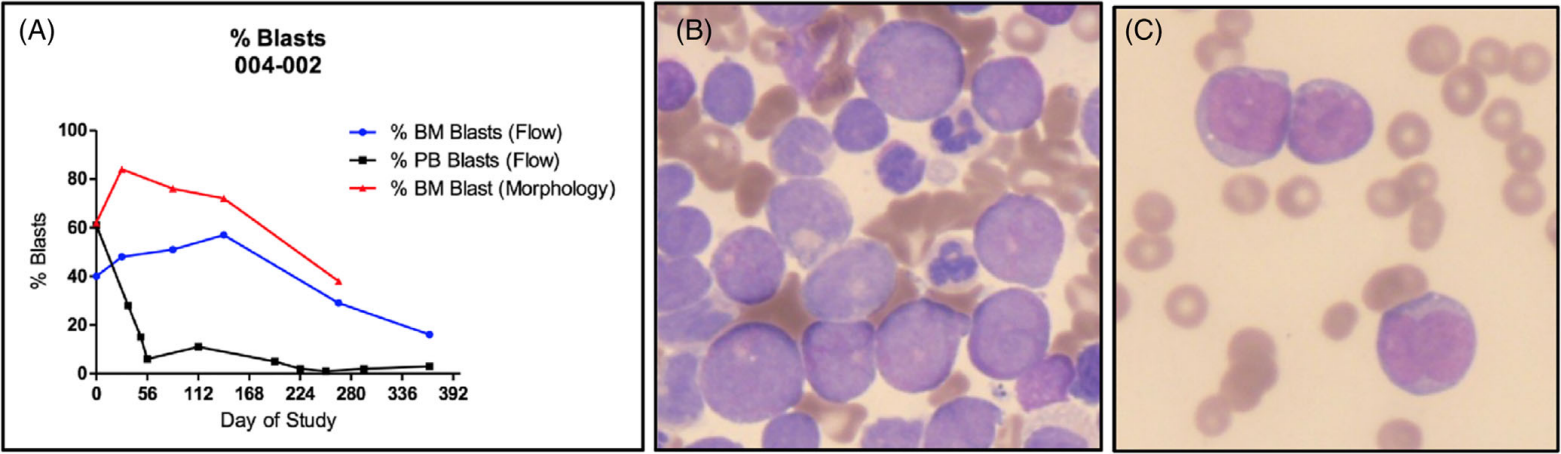
(A) Percentage of blasts in serial peripheral blood and bone marrow samples. (B) AML (FAB M2) on February 2017 bone marrow aspirate. (C) Monoblasts in November 2017 bone marrow

**FIGURE 3:**
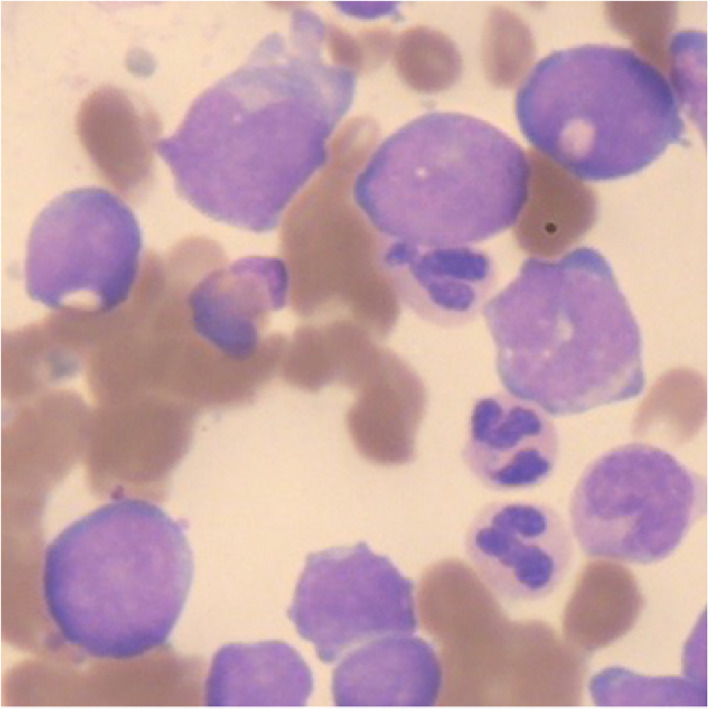
Bone marrow aspirate at 6 months on Bomedemstat

LSD1 inhibitor, Bomedemstat, was commenced at an increasing dose of 3 mg/kg in 14 day cycles following enrolment in a clinical trial. The initial blast response was remarkable, with peripheral blast counts falling from 15.49 × 10^9^/L to <1.00 × 10^9^/L by the end of cycle 2. Peripheral blood and three monthly marrows showed reducing blast counts with a rising percentage of monoblasts. This was noted on morphology and supported by flow cytometry (Figure 1), indicating differentiation of blasts from an immature phenotype down the monocytoid pathway with reduction in blasts (Figures 2 and 3), in response to LSD‐1 inhibition. Supportive transfusions of packed red cells and platelets were continued intermittently and she was admitted to hospital for febrile illness requiring intravenous antibiotics on four occasions while on study, each time demonstrating a neutrophil response and recovering.

Bomedemstat was mostly well tolerated, with the only direct side effect being gastrointestinal symptoms when dose was increased to 6 mg/kg at C10, which resolved on dose reduction back to 3 mg/kg. Combination therapy with all‐*trans* retinoic acid (ATRA) was given for 3 months, however ceased due to dry skin and eyes and desquamation of feet. The patient was admitted to hospital for febrile illness requiring intravenous antibiotics on four occasions while on Bomedemstat, recovering each time and demonstrating a peripheral blood neutrophil response. She also developed Sweet's syndrome (neutrophilic dermatitis) after 15 months on therapy, which resolved with steroid treatment. In the setting of neutropenia, 26 months after being enrolled on study, the patient developed urosepsis and osteomyeltis, withdrawing from active therapy prior to demise.

## 3 DISCUSSION

This case demonstrates an outstanding response to treatment with the LSD1 inhibitor Bomedemstat, with a rapid reduction of blast counts with increased monocyte differentiation. This response was sustained with minimal complications. Considering the typically poor prognosis in secondary AML (in older adults reported to be a median of only 2 months)^1^ and limited therapeutic options available, this patient demonstrates a significant survival benefit from treatment with an LSD1 inhibitor.

Identification of driving genetic abnormalities such *JAK2*, *MPL* and *CALR* mutations in MPN, have helped classify as well as herald targets for therapy.^2^ Other than JAK‐2, non‐driver mutations have been identified in about half of essential thrombocytosis (ET) cases (the most frequently seen are Ten‐Eleven Translocation 2 [*TET2*] and *ASXL1*),^3^ demonstrating inferior overall survival, leukaemia free survival, and myelofibrosis (MF) free survival. LSD1 is a component of the transcription repressor complex CoREST, and has a critical role in the self‐renewal, proliferation and differentiation of haematopoietic stem cells.^4^ LSD1 inhibitors act by disrupting the interaction between LSD1 and the transcription factor GFI1B on chromatin.^5^ Recently, LSD1 has been shown to be essential for glycosis and haeme synthesis, stabilising with GATA1, and shaping metabolic phenotypes in erythroid leukaemia cells.^6^

A number of LSD1 inhibitors have been developed and are in clinical trials for patients with AML and MPNs. LSD1 inhibitors act by disrupting the interaction between LSD1 and the transcription factor GFI1B on chromatin. It has been demonstrated that LSD1 enzyme activity is not required for AML survival, and the main mechanism of action is through protein binding interactions.^7^ Similarly, knockout of LSD1 in AML in murine models led to blast differentiation with monocytic features, increased ATRA sensitivity, and prolonged survival, associated with upregulation of GFI1B and IRF8 (monocytic transcription factor).^8^ The LSD1 inhibitor Bomedemstat (IMG‐7289) demonstrated sustained clinical activity and a favourable tolerability in advanced MF.^9^ Similarly, the LSD1 inhibition have been associated with some encouraging outcomes in the treatment of AML.^10^

Our case with secondary AML transformed from ET, had a prolonged response to LSD1 inhibition with Bomedemstat. The monocytoid changes seen morphologically were confirmed by flow cytometry, by gating and surface markers. Monocyte expansion following commencement of therapy was seen in this patient. LSD1‐deficient animals show inhibition of granulopoiesis and stimulation of monocytopoiesis,^11^ and knockout of LSD1 in AML murine models has led to blast differentiation with monocytic features.^8^ These findings and this patient's response to treatment with an LSD1 inhibitor suggests that LSD1 plays an important role in lineage choice during granulocyte differentiation and maturation.^12^

LSD1 inhibition leads to increased sensitivity of AML blasts to ATRA.^13^ In this case, ATRA was used in combination with Bomedemstat for three months, however ceased due to side effects, so the impact of ATRA in this specific leukaemia is unclear. Treatment with an LSD1 inhibitor in combination with Ruxolitinib may also be of interest for future studies of patients with secondary AML transformed from *JAK2* positive ET, as it has demonstrated synergy in murine models for MF.^14^ The LSD1 inhibitor Bomedemstat has received fast‐track designation for the treatment of myelofibrosis.^15^

## 4 CONCLUSION

Targeting of IDH1 with its integral cellular function has advanced understanding and possible treatments in haematological malignancies. This case demonstrates impressive prolonged response in secondary AML, arising from ET, to the LSD1 inhibitor Bomedemstat. In addition to its effective role in the treatment of myelofibrosis,^5^ this illustrates the potential role for novel targeted therapies, alone or in combination in this group of neoplasms. This strategy may have a special role in treatment of specific AML subtypes, including secondary leukaemias arising from MPN.

## CONFLICT OF INTEREST

The authors have stated explicitly that there are no conflicts of interest in connection with this article.

## AUTHOR CONTRIBUTIONS

**Samantha Hodges:** Data curation (equal); formal analysis (equal); methodology (equal); writing – original draft (equal); writing – review and editing (equal). **Julian Cooney:** Data curation (equal); formal analysis (equal); methodology (equal); project administration (equal); writing – review and editing (equal).

## ETHICS STATEMENT

Signed consent was obtained by the patient prior to demise.

## Data Availability

The data that support the findings of this study are available from the corresponding author, [JC], upon reasonable request.
